# Effect of Luting Cement Film Thickness on the Pull-Out Bond Strength of Endodontic Post Systems

**DOI:** 10.3390/polym13183082

**Published:** 2021-09-13

**Authors:** Khalil Aleisa, Syed Rashid Habib, Abdul Sadekh Ansari, Ragad Altayyar, Shahad Alharbi, Sultan Ali S. Alanazi, Khalid Tawfik Alduaiji

**Affiliations:** 1 Department of Prosthetic Dental Sciences, College of Dentistry, King Saud University, Riyadh 11545, Saudi Arabia; kali@ksu.edu.sa; 2 Assistant Consultant in Prosthodontics, Dental University Hospital, King Saud University Medical City, Riyadh 11545, Saudi Arabia; drsadeq.ans@gmail.com; 3 Dental Intern, College of Dentistry, King Saud University, Riyadh 11545, Saudi Arabia; Ragadstayyar@gmail.com (R.A.); Shahadalhar@gmail.com (S.A.); 4 Restorative Resident, Ministry of Health, Riyadh 11153, Saudi Arabia; salraslani1@gmail.com; 5 General Dentist, Ministry of Health, Riyadh 11545, Saudi Arabia; Aldaaigi1992@gmail.com

**Keywords:** endodontics, posts, dowel, fiber posts, prefabricated posts, titanium posts, pull-out bond strength, cement, thickness

## Abstract

Optimal bond strength between the prefabricated post/dowel to the surrounding dentin is essential. The present study aimed to analyze and compare the effect of three different cement film thicknesses on the pull-out bond strength of three different prefabricated post systems. Extracted natural teeth (N = 90) with similar root dimensions were acquired. Teeth were mounted in resin blocks, endodontically treated, sectioned at cemento-enamel junction, divided into three groups (A: Parapost Fiber Lux plus; B: 3M ESPE Relyx fiber post; and C: Parapost XP), and stored. Uniform post spaces were prepared for the groups (A and C: Length = 8 mm, Width = 1.5 mm; B: Length = 8 mm, Width = 1.6 mm). Each group (N = 30) was further subdivided into three subgroups (n = 10) based on the size (4, 5, and 6) of the post and cemented with resin cement (MultiLink-N, Ivoclar Vivadent). After thermocycling, the specimens were subjected to a pull-out test using a universal testing machine, and tensile force was recorded (MPa). Digital microscopic evaluations were performed for modes of failure. ANOVA and Tukey-HSD tests were used for statistics. Significant differences were observed for each tested material (*p* = 0.000). The lowest and highest bond strength values were recorded for Group C (Titanium post) and Group A (000), respectively. Multiple comparisons showed significance (*p* < 0.05) among all the groups, except for space 1 and space 2 (*p* = 0.316) for Group A. Most of the failures occurred within the cement-dentin and post-cement interface (Adhesive failures, 73.5%). An increase in the luting cement film thickness results in the decrease in pull-out bond strength of prefabricated posts luted with resin cement, irrespective of the type/material/shape of the post. The serrated fiber posts showed the highest pull-out bond strength compared to the smooth surfaced fiber posts or serrated metal posts. Increased pull-out bond strengths were observed when appropriate post space was created with the same sized drill as the post size.

## 1. Introduction

Restoration of endodontically treated teeth with extensive loss of coronal tooth structure usually requires the placement of a post inside the root canal to increase retention and stability of the final restoration [1]. Recently, most of the clinical procedures use prefabricated posts adhesively cemented to root canal dentin due to the numerous advantages of prefabricated posts over custom posts. Various post and core systems, a variety of materials, and several methods are used to restore endodontically treated teeth. Although there is increasing demand for using adhesively bonded prefabricated posts, which have the advantages of increased retention, aesthetics, and reinforcement of tooth structure, many factors that may affect the outcome must be considered [2]. Anatomical variations of the roots and the shape of the root canal significantly influence the post placement and cementation inside the root canal; therefore, some alteration of the natural shape of the canal is necessary to adapt a post inside the root canal. Additionally, the material, shape, and size of the post; the polymerization process of the luting cements; and cement thickness are factors that can influence the adhesion of endodontic posts to root canal dentin [3].

Numerous studies have shown the clinical survival and failure of teeth restored with posts and cores. Different failure modes include loss of retention of the post, root fractures, and fracture of the post or the core; these studies reported the loss of retention of posts as one of the most frequent types of failure [4]. Rasimick et al. [5], in their review, found that debonding loss accounted for 37% of all reported failures in adhesively luted posts. These authors also calculated pooled odds of 2.3% that a restoration would fail due to debonding of posts luted with adhesive resin cements, compared with the 4.3% calculated for posts luted with zinc phosphate or glass ionomer cement [4,5]. 

The factors related to the post and the luting cement, as well as the cement-post and cement-dentin interactions and film thickness of the cement may also influence the retention of posts [6]. A uniform cement film thickness of 25 and 50 microns has been accepted for luting fixed restorations. To obtain optimal and uniform thickness of the cement, a precise fit of the post in the post space is required [7] but, due to the irregularities of the root canal morphology or the tapered endodontic preparations, obtaining a precisely fitting post and a uniform film thickness often require extensive post-space preparation that may lead to weakening of the remaining tooth structure, compromising the longevity of endodontically treated teeth [8]. Therefore, clinicians need to balance a perfect fit of the post inside the root canal with the amount of internal root dentin to be removed. 

The retention of prefabricated posts is also influenced by the material and shape of the post [9]. Several modifications have been made to the design of the posts to improve physical properties; these include surface modifications and different shaped posts [9]. Different types of surface treatments have been recommended for increasing the retention of posts. Proper cementation technique with good choice of luting material enhances the bond strength of these posts, which increases the longevity of the final restorations [10]. Numerous cementation techniques and materials have been proposed, starting with the use of conventional cements and, lately, resin based cements. Little information is available on the role of cement film thickness and its effect on the retention of posts [6,9,10,11]. Previous clinical retrospective studies have demonstrated that the debonding of posts occurs when luting cement film thickness is increased [12,13,14]. However, there is no consensus on the ideal thickness of the luting cement to improve the retentive bond strength of posts in endodontically treated teeth [15]. 

Additionally, polymers play a major role in most areas of preventive and restorative dentistry. Specifically, in endodontic science, resin cements, sealers, gutta-percha, composite resins, and fiber-reinforced posts are being used extensively. The use of polymers is becoming more common due to their improved physical, mechanical, and biological properties [16].

The present study was conducted with the purpose of analyzing the effect of three different cement film thicknesses on the pull-out bond strength between three post systems and root dentin. The null hypotheses of the present study assumed that (i) different cement film thicknesses around the posts have no effect on the retentive bond strength of posts, (ii) the type of luting cement would have no effect on the retentive bond strength of posts, and (iii) and different post systems would not affect the bond strength of endodontic posts.

## 2. Materials and Methods

### 2.1. Sample Size and Sampling Technique

Ninety freshly extracted, caries-free, single-rooted human mandibular first premolar teeth with similar root lengths were selected for this study. At alpha 0.05, with the power of 0.90, and the effect size of 0.5, the total sample size for testing the pull-out bond strength of three post material groups with three cement film thicknesses was 90. The teeth were obtained from young adults extracted for orthodontic reason. The Schneider [16] method was applied to select teeth with single, non-calcified, and straight canals. All the selected teeth were subjected to radiographic examination from the buccolingual and mesiodistal directions using a dental X-ray machine (CS2100, Carestream Dental, Kodak, Rochester, NY, USA). The selected teeth were cleaned using an ultrasonic cleaner, disinfected with 5.25% NaOCl solution, and stored in antimicrobial preservative container of aqueous solution of 0.5% Chloramine T (Delchimica Scientific Glassware, Napoli, Italy) and used within 6 weeks of extraction. The selected teeth were then mounted in autopolymerizing acrylic resin blocks (Ortho-Resin, DeguDent GmbH, Hanau Hessen, Germany) of 2 cm diameter, exposing the anatomic crown and 2 mm of coronal root portion.

### 2.2. Specimen Preparation

**Figure 1:**
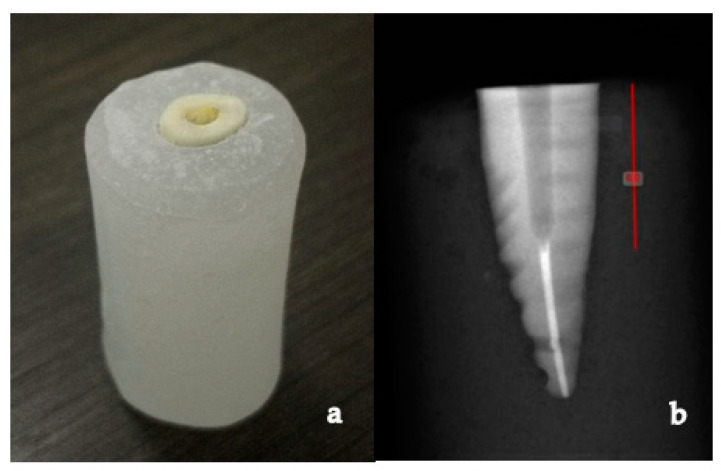
(**a**) Mounted specimen sectioned the cement-to-enamel junction; (**b**) Radiograph of optimally prepared post space.

**Figure 2:**
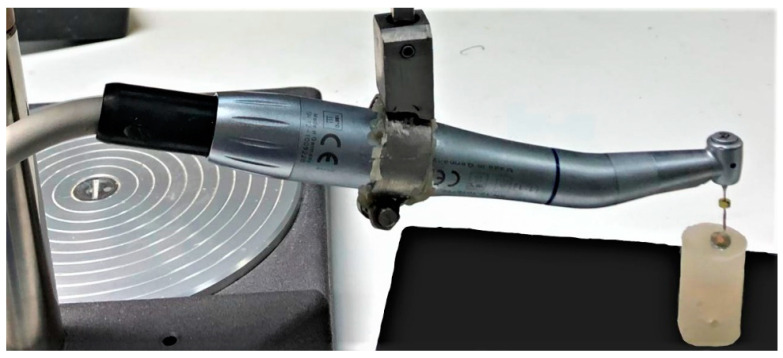
Standardized post space preparation with customized hand piece attached to dental surveyor.

A double sided diamond disc (NTI Sintered, Kerr Corporation, Brea, CA, USA) with a high-speed dental air-turbine handpiece (NSK, Nakanishi Inc. Shinohinata, Kanuma Tochigi, Japan) was used to section the teeth at the level of the cement-to-enamel junction to remove the coronal part of the teeth, under copious water irrigation (Figure 1a). Pulpal tissues were extirpated using a barbed broach (Dentsply Maillefer, Tulsa, OK, USA). Working length was determined with a size 10 K-file (Dentsply Maillefer, Tulsa, OK, USA). The root canals were cleaned, shaped as per the crown-down technique of biomechanical preparation using K-files sizes 15 and 20 (Dentsply Maillefer, Tulsa, OK, USA), followed by Protaper Ni-Ti rotary instruments (sizes S1, S2, F1, F2; Dentsply Maillefer, Tulsa, OK, USA) in a high torque endodontic motor (X-Smart, Dentsply Maillefer, Tulsa, OK, USA) at 350 rpm using the crown-down technique to the full working length. NaOCl (5.25%) was used for irrigation after each file. The prepared root canals were obturated with gutta-percha (Kerr Corporation, Brea, CA, USA) and AH plus sealer (Dentsply, Maillefer, Tulsa, OK, USA) using the warm vertical condensation method. Coronal 2 mm of gutta-percha was removed with a gates glidden drill, and the access opening was sealed with temporary restorative material (Cavit, 3M ESPE, St. Paul, MN, USA) [17]. All the teeth were then stored in 100% relative humidity at room temperature [18]. After 24 h of obturation, the specimens were prepared for post space by using peeso reamers (Pulpdent Corp, Watertown, MA, USA) size 4 and 5 under copious water irrigation with a slow speed dental handpiece (NSK, Nakanishi Inc. Shinohinata, Japan) attached to a customized dental surveyor (J. M. Ney Co., Hartford, Connecticut, CT, USA) to guide post space preparation parallel to the long axis of the teeth (Figure 2).

All the specimens were divided into three main groups (N = 30, Groups A, B, and C) according to the type of post used, and each main group was further subdivided into three subgroups (n = 10, Subgroups A1, A2, A3, B1, B2, B3, C1, C2, C3) according to the size of post used, as shown in Table 1. All the specimens from Group A were finally prepared for uniform post spaces of 8 mm in length and 1.5 mm in diameter using Parapost drill size 6 (P-42-6, diameter 1.5 mm, Black, Coltene/whaledent Inc. Feldwiesenstrasse, Altstätten, Switzerland), leaving a minimum of 5 mm of apical gutta-percha as an apical seal.

All specimens from Group B were finally prepared for uniform post spaces of 8 mm length and 1.6 mm diameter using a 3M ESPE fiber post drill, size 2 (diameter 1.6 mm, Red, 3M ESPE, St. Paul, MN, USA), leaving a minimum of 5 mm of apical gutta-percha as an apical seal (Figure 1b). Similarly, all specimens from Group C were finally prepared for uniform post spaces of 8 mm length and 1.5 mm diameter using Parapost drill size 6 (P-42-6, diameter 1.5 mm, Black, Coltene/whaledent Inc. Feldwiesenstrasse, Altstätten, Switzerland), leaving a minimum of 5 mm of apical gutta-percha as an apical seal.

A new drill was used for every 10 specimens. Post spaces were cleaned with 5.25% NaOCl (Ogna, Milan, Italy) and 17% EDTA (Pulpdent, Watertown, MA, USA) to remove traces of gutta-percha and sealer. The details of the materials and instruments used are presented in Table 2. Periapical radiographs were taken for all specimens to ascertain that no gutta-percha residues remained in the post space and there was no perforation (Figure 1b).

**Figure 3:**
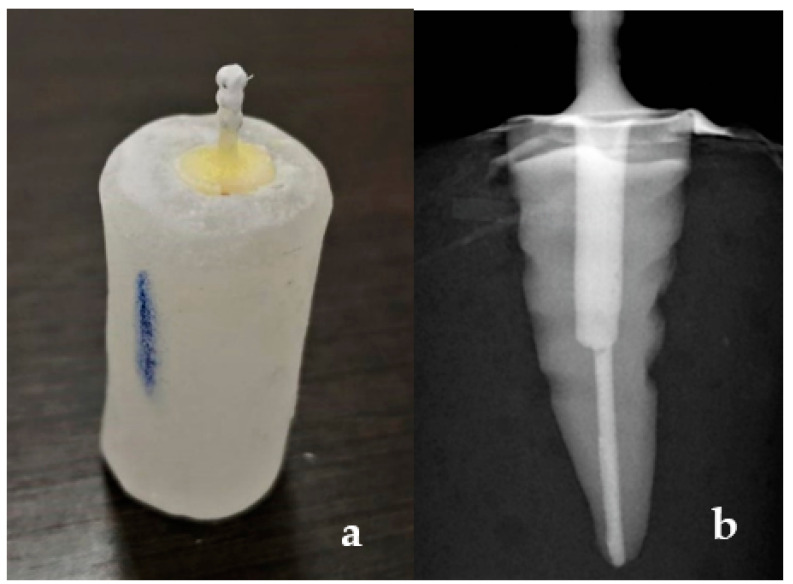
(**a**) Cemented fiber post; (**b**) Radiograph after the cementation of post.

Post spaces of specimens from each group were etched with 37% Orthophosphoric acid etching gel (DentoEtch, Itena-Clinical, Villepinte, France) for 5 s, then cleaned with normal saline and dried using absorbent paper points (Roeko paper points; Coltene/Whaledent, Cuyahoga Falls, OH, USA). Each post was cemented into the respective specimens using Multilink N (Ivoclar Vivadent AG, Schaan, Liechtenstein) dual cure self-adhesive resin cement according to the manufacturer’s instructions. The posts were cleaned with alcohol and then Monobond N (Ivoclar Vivadent AG, Schaan, Liechtenstein) was applied to the posts and dried for 60 s, as recommended by the manufacturer. The prepared canals were coated with Multilink primer using a microbrush, and any excess was blotted with absorbent paper points. The resin cement was placed in the root canals with an elongation tip. Posts were then coated with resin cement and inserted into the prepared canals with finger pressure. Dental Surveyor (J. M. Ney Company, Hartford, Connecticut, CT, USA) was used to guide the cementation of posts parallel to the long axis. Excess cement was removed, and the specimen was cured with polymerization light (XL 2500; 3M ESPE, St. Paul, MN, USA) for 20 s (Figure 3a). Post cementation periapical radiographs were recorded for verification of the post fit (Figure 3b).

### 2.3. Measurement of Cement Film Thickness

The diameter of three randomly selected posts from each group was measured by a digital caliper (U1025, Mitutoyo, Kawasaki, Japan). Three measurements per post were made, 8 mm from the apical end, and a final mean diameter was calculated. For Groups A and C, the mean diameter of post sizes 4, 5, and 6 were obtained as 0.9 mm ± 0.05 mm, 1.15 mm ± 0.05 mm, and 1.4 mm ± 0.05 mm, respectively. For Group B, the mean diameter of post sizes 0, 1, and 2 were obtained as 1 mm ± 0.05 mm, 1.2 mm ± 0.05 mm, and 1.5 mm ± 0.05 mm, respectively. The diameter of each prepared post space was then measured under a digital microscope (HIROX, KH-7700, Digital microscope system, Tokyo, Japan) at 50× magnification at the coronal portion of the prepared canals. Four measurements were made per canal. Mean values and standard deviations of the post space diameters were then calculated from the preparations performed with the Parapost drill size 6 for Groups A and C and 3M fiber post drill size 2 for Group B. The obtained mean post space diameters for Groups A and C were 1.5 mm ± 0.05 mm, and for Group B they were 1.6 mm ± 0.05 mm. Cement film thickness was calculated by subtracting the mean diameter of the posts from the mean diameters of the post spaces, as shown below.

Cement film thickness (mm) = (Diameter of post space (mm) − Diameter of Post (mm))/2 [6].

The calculations resulted in the following three mean cement film thicknesses for all the groups: For post space 1—0.6 ± 0.05 mm (600 µm), for post space 2—0.35–0.4 ± 0.05 mm (350–400 µm), and for space 3—0.1 ± 0.05 mm (100 µm), as shown in Table 3. 

### 2.4. Thermocycling and Testing of Specimen

**Figure 4:**
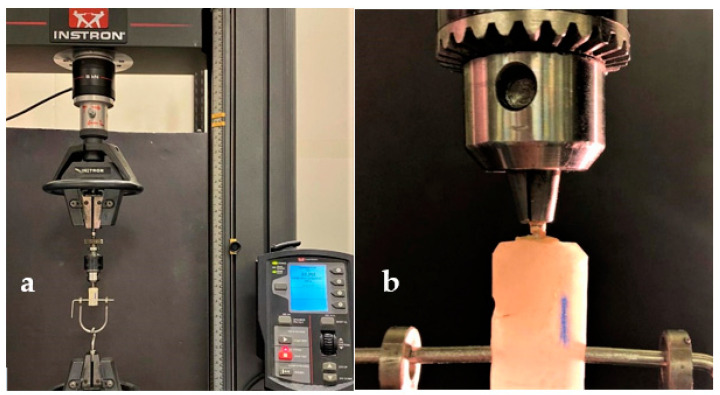
(**a**) Samples attached to the Instron testing machine; (**b**) Close up view of the pneumatic grips that grasped the post head and pulled the post along its long axis.

After cementation, all the specimens were thermocycled (TC 50 C/550C, 5000 cycles) and stored in 100% relative humidity at room temperature for 24 h before testing. The specimens were subjected to a pull-out test using a universal testing machine at a crosshead speed of 0.5 mm/min, and the maximum force required to dislodge each post was recorded. Each tooth specimen was vertically secured in the universal testing machine (Instron, model 8500 Plus; Dynamic Testing System; Instron Corp). A tensile force was applied to dislodge the post by using pneumatic grips that grasped the post head and pulled the post along its long axis (Figure 4). A constant loading rate of 0.5 mm/min was applied until failure occurred. The peak force recorded at the point of extrusion of the post from the tooth was considered the point of bond failure. Tensile force was recorded in megapaskal (MPa).

### 2.5. Microscopic Evaluation and Failure Mode

**Figure 5:**
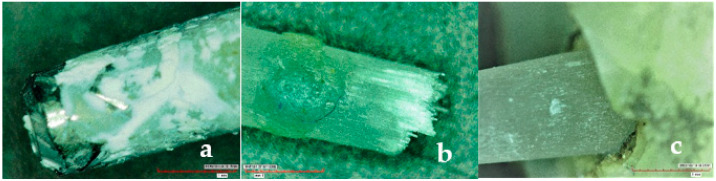
(**a**) Specimen with adhesive failure; (**b**) Specimen with cohesive failure; (**c**) Specimen with mixed type failure.

The fractured specimens were observed under digital microscope (HIROX, KH-7700, Digital microscope system, Tokyo, Japan) at 50× magnification to evaluate the modes of failure (Figure 5). All specimens were divided into three groups based on the mode of failure: cohesive (fracture within the post or the cement), adhesive (Fracture at the cement-dentin interface), and mixed failures (fracture extending to the dentin through luting cement, complex fractures). All specimens were analyzed by three dental specialists trained in microscopic analysis to standardize the mode of failure. Based on the failure modes, the percentage of failed specimens were calculated and recorded.

### 2.6. Statistical Analysis

The statistical package for social sciences (SPSS, version 22, Chicago, IL, USA) was used for the statistical analysis. The mean and standard deviations of pull-out bond strengths were calculated for all three test groups with the three post spaces tested. The normality of the data was tested with Shapiro–Wilk test and was found to be normal. Using One-way ANOVA and post-hoc Tukey’s HSD test, the data was analyzed. Descriptive statistics for the failure mode data was also completed. The accepted level of error was set at *p* < 0.05.

## 3. Results

In this present in vitro research, the effect of three different cement film thicknesses on the pull-out bond strength (Tensile stress) of three commonly used prefabricated post systems luted to the root dentin was analyzed.

### 3.1. Analysis of Pull-Out Bond Strength

The measurements for all the tested group specimens were recorded in megapascals (MPa). Table 4 shows the mean, standard deviation, minimum, maximum values, and ANOVA results for the bond strength of all three tested group materials with three different post spaces. With regards to the three different cement spaces, statistically significant differences were observed for each type of tested group materials (*p* = 0.000).

The lowest bond strength values were recorded for the Group C (Titanium post) material, followed by the Group B (Fiber post tapered) material, and the highest bond strength values were recorded for the Group A (Fiber post parallel serrated) material (Table 4). 

Table 5 presents the mean difference and post-hoc Tukey’s test values between the bond strength of three cement spaces for three types of post materials. All of the mean differences showed significance (*p* < 0.05), except the mean difference between space 1 and space 2 (*p* = 0.316) for the Group A post material (Table 5).

The descriptive statistics and ANOVA results for the same post space with the three types of post materials is displayed in Table 6, which shows significant differences (*p* = 0.000) between the bond strength values for the three different types of post systems (Table 6).

### 3.2. Analysis of Failure Modes

**Figure 6:**
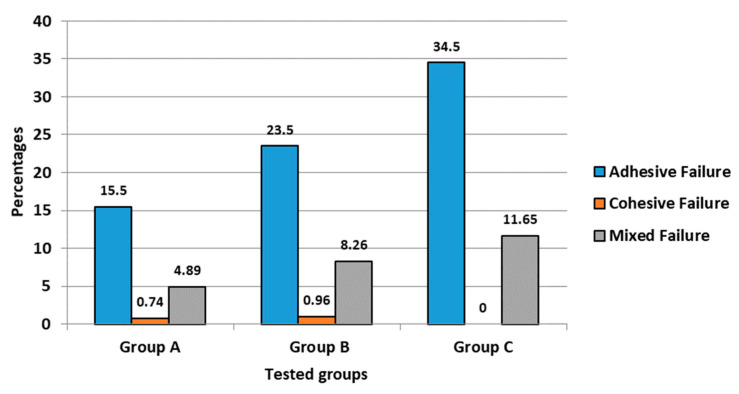
Analysis of failure modes for the three tested groups.

The analysis of failure modes (Figure 6) revealed that most of the failures occurred within the cement-dentin and the post-cement interface (adhesive failures, 73.5%), with some of the specimens having mixed failures within the dentin-cement-post assembly (mixed failures, 24.8%); very few specimens depicted fracture within the post (cohesive failures, 1.7%).

## 4. Discussion

In the present study, the effect of three different cement film thicknesses on the pull-out bond strength of endodontic post systems on natural teeth was evaluated. In this study, three different post systems with three different sizes of posts were used, while the post space diameter remained uniform, and the same resin cement was used for all the specimens tested.

In previous studies, different methods, such as pull-out; push-out, and micro-tensile tests, have been used for the evaluation of the bond strength of posts cemented to natural tooth roots. The pull-out tensile bond strength testing of the cemented posts is considered superior to the other tests [17,18,19]. Previous studies have also used pull-out bond strength on natural teeth to clinically simulate the retention of endodontic post systems, and they have reported similar results [20,21]. These retrospective clinical studies have reported that most common failures of endodontic post is the pull-out of the post, cement, or the cement restoration assembly [22]. The retention of a post to the teeth is dependent on a number of factors, such as the type of cement used for luting, the cementation bond between the cement-post and cement-dentine, the mechanical/physical properties of the posts and cements used, the influence of water sorption on contraction/hydroscopic expansion of resin cements, and the shape (parallel sided, tapered) and surface texture (smooth, serrated, grooved and/or threaded) of the post [23,24,25]. Therefore, based on these observations, in this study, investigation and comparison of the tensile bond strength of cemented prefabricated smooth, serrated, fiber posts and metal posts under standardized conditions was performed/preferred.

From the results of this study it is evident that statistically significant differences (*p* = 0.000) were observed for each tested group; therefore, the null hypothesis was rejected. The lowest bond strength values were recorded for Group C (Paracore Xp-titanium post system), and the highest pull-out bond strength values were observed for Group A (Paracore fiber lux-Fiber post system). This shows that bond strengths are affected by the type of post material used. The explanation for this finding may be that the retention of posts in the endodontically treated teeth depends on numerous factors such as size, shape, surface characteristics, and the material of endodontic posts. Similar findings have been observed in numerous previous studies that reported posts with a dentine-like low elastic modulus, such as fiber-reinforced posts cemented adhesively with resin cements, showed good performance and high retention compared to posts with a high elastic modulus, such as titanium or ceramic posts [13,14,15,16,17].

According to research studies [9,10], defects in the microstructure are present randomly in all materials. However, the initiation of cracks in these micro defects occurs only when excess load is applied. Therefore, it can be concluded that, with the increase in the thickness of the luting cement, the chances of micro defects are more, and the probability of crack initiation and propagation are high [22]. This will lead to the decrease in the resistance of thicker cement layers. Due to the variations in the shape and size of the root canals, the precise and accurate fitting of the prefabricated posts to the prepared post space, most of the times, is impossible [26]. Hence, extensive preparation of the root canal should be avoided, which may result in the weakening of the tooth. This inevitably results in variations of the cement thickness in the root canals. To optimize retention in these cases, and in cases of evidently oversized post spaces, the posts should be luted adhesively with resin cement, which effectively bonds to the post as well as to the dentin [16].

When the effect of cement film thickness on pull-out bond strength is compared, the results show significant difference between the groups studied (*p* < 0.05); the highest bond strength was observed for Space 3 (size 6 post luted in post space prepared with size 6 Parapost drill). This means that the cement film thickness is minimal (100 μm) when the same sized drill is used, corresponding to the post size, as recommended by the manufacturer. The lower bond strength values were recorded when small sized posts were used in oversized post spaces, as in space 1 (600 μm) and space 2 (350–400 μm) (size 4 and size 5 posts luted in post space prepared with size 6 Parapost drill). These findings are similar to previous studies, which showed that the debonding of posts occurs when cement film thickness is increased [12,14]. It was proposed from the studies that when the cement layer is thinner, there will be less micro-porosities and polymerization stress, leading to higher retentive bond strength [14,18]. 

Logically, and according to the results of this study, the fiber posts showed better pull-out resistance compared to metal posts due to their bonding ability to the resin cement. An additional factor that may have contributed to the better pull-out resistance could be the effect of post surface treatment on the bond between the post and the dentine. The surface treatment of the posts increases the bond strength of the fiber posts [10,27]. However, according to the results, it was observed that there was a decrease in the retention of posts with the increase in the luting cement film thickness, irrespective of the type or make of the posts. Possibly, some variables, such as the filler content of the resin cement used, could have an effect on the results of the present investigation. Some of the studies have reported that the filler size of the composite resin can alter both adhesive [28] and mechanical [29,30] properties. Therefore, further reports are needed in order to explore the filler content of the polymer resin cement used. 

The analysis of failure modes under digital microscope revealed that the pre-dominant failure types in the groups were adhesive failures (73.5%) between the post-to-cement interface and the cement-to-dentin interface. Mixed failures (resin cement covering from 50% to 100% of the post diameter) accounted for about 24.8%. Similar findings were reported by Balbosh and Kern [11]. However, Rasimick et al. [5] reported that the adhesive failure between the dentin and the adhesive resin was a pre-dominant failure. Although a high percentage of specimens failed adhesively during the test, microscopic observations confirmed that either luting cement, root dentin, or the fiber post were all likely to fracture considering that the load applied to the assembly (dentin, cement, and post) generated stress concentration at the cement-to-dentin and the cement-to-post interfaces. Similarly, FEA analysis by Prisco et al. [23] also demonstrated that the stress concentration at the cement-to-post interface is likely to initiate debonding through brittle crack propagation from the top of the cement layer, downward along the post surface. Additionally, the deformation of the posts under tensile load might have created some additional resistance to its dislodgment, thereby increasing stress concentration inside the luting cement and root dentin, leading to mixed fractures. Furthermore, Le Bell and Lassila et al. [26] reported that when endodontic posts are perfectly bonded, more stress is transferred to the dentin instead of being concentrated at the interface, thus explaining the multiple failure modes observed.

The study was performed with uniform post spaces, using three different sizes of prefabricated posts for each system, and an attempt was made to standardize conditions during the specimen preparation. However, differences in the results of the present study may have been caused by limitations such as the inherent characteristics of the adhesive system used and the experience and skills of the operators. Additionally, the inherent characteristics of the root dentin, high 𝐶-factor, the presence of a smear layer, difficult access for instrumentation and light polymerization, the presence of the remnants of gutta-percha and endodontic sealer cement, could all have influenced the results of this study. However, all possible measures were taken to ensure standardization. Nevertheless, the results of the present study should be interpreted and applied cautiously as the present study was performed on human extracted teeth that may have lost their elasticity due to aging, resulting in variations in the bond strength of the specimens. There could also be variations in the post space preparations or cementation due to human error. Therefore, further studies, which include artificial aging to evaluate the effects of the thermal and mechanical aging of human tooth, while using more accurate methods to measure the cement film thickness, are suggested.

## 5. Conclusions

From the results of the present study, the following conclusions can be drawn:

1. The choice of post material has an effect on the pull-out bond strength, with fiber posts showing better pull-out bond strength compared to metal posts.;
2. An increase in the luting cement film thickness results in the decrease in pull-out bond strength of the posts luted with resin cement, irrespective of the type/material/shape of the post;
3. The serrated fiber posts showed the highest pull-out bond strength compared to the smooth surfaced fiber posts or serrated metal posts;
4. Increased pull-out bond strengths were observed when appropriate post space was created with the same sized drill as the post size.

## Figures and Tables

**Table 1:** Details of the tested post materials used for different groups in the research study.

Group (N-30)	Subgoup (n-10)	Post Material	Trade Name	Manufacturer	Post Size	Lot Number
**Group-A**	A1	Fiber post parallel serrated (For post space 1)	Parapost Fiber Lux plus	Coltene/Whaledent Inc.	Size 4, diameter 0.9 mm Yellow, PF1714	H65570
	A2	Fiber post parallel serrated (For post space 2)	Parapost Fiber Lux plus	Coltene/Whaledent Inc.	Size 5, diameter 1.15 mm Red, PF1715	H65570
	A3	Fiber post parallel serrated (For post space 3)	Parapost Fiber Lux plus	Coltene/Whaledent Inc.	Size 6, diameter 1.4 mm Black, PF1716	H65570
**Group-B**	B1	Fiber post tapered (For post space 1)	3M ESPE Relyx fiber post	3M ESPE	Size 0, diameter 1.0 mm White	311980506
	B2	Fiber post tapered (For post space 2)	3M ESPE Relyx fiber post	3M ESPE	Size 1, diameter 1.2 mm Yellow	311980506
	B3	Fiber post tapered (For post space 3)	3M ESPE Relyx fiber post	3M ESPE	Size 2, diameter 1.5 mm Red	311980506
**Group-C**	C1	Titanium post parallel serrated (For post space 1)	Parapost XP	Coltene/Whaledent Inc.	Size 4, diameter 0.9 mm Yellow, P-784-4	H17858
	C2	Titanium post parallel serrated (For post space 2)	Parapost XP	Coltene/Whaledent Inc.	Size 5, diameter 1.15 mm Red, P-784-5	H17858
	C3	Titanium post parallel serrated (For post space 3)	Parapost XP	Coltene/Whaledent Inc.	Size 6, diameter 1.4 mm Black, P-784-6	H17858

**Table 2:** Details of the materials and instruments used for preparation of the samples.

	Material	Trade Name	Manufacturer	Size	Lot Number
**Cement**	Dual cure resin cement	Multilink N	Ivoclar Vivadent	-	X29755
**Parapost drill**	Titanium	Parapost XP	Coltene/Whaledent Inc.	Size 6 diameter 1.5 mm Black, P426	H65570
**3M fiberpost Drill**	Stainless steel	3M ESPE fiber post drill	3M ESPE	Size 2, Diameter 1.6 mm Red	985627

**Table 3:** Measurement of cement film thickness.

S. No.	Subgroup	Post Type	Mean Post Diameter (mm)	Mean Post Space Diameter (mm)	Mean Cement Film Thickness (mm)
1.	A1	Fiber post parallel serrated (size 4, For post space 1)	0.90 ± 0.05	1.50 ± 0.05	0.60 ± 0.05 (600)
	A2	Fiber post parallel serrated (size 5, For post space 2)	1.15 ± 0.05	1.50 ± 0.05	0.35–0.40 ± 0.05 (350–400)
	A3	Fiber post parallel serrated (size 6, For post space 3)	1.40 ± 0.05	1.50 ± 0.05	0.10 ± 0.05 (100)
2.	B1	Fiber post tapered (size 0, For post space 1)	1.00 ± 0.05	1.60 ± 0.05	0.60 ± 0.05 (600)
	B2	Fiber post tapered (size 1, For post space 2)	1.20 ± 0.05	1.60 ± 0.05	0.35–0.40 ± 0.05 (350–400)
	B3	Fiber post tapered (size 2, For post space 3)	1.50 ± 0.05	1.60 ± 0.05	0.10 ± 0.05 (100)
3.	C1	Titanium post parallel serrated (size 4, For post space 1)	0.90 ± 0.05	1.50 ± 0.05	0.60 ± 0.05 (600)
	C2	Titanium post parallel serrated (size 5, For post space 2)	1.15 ± 0.05	1.50 ± 0.05	0.35–0.40 ± 0.05 (350–400)
	C3	Titanium post parallel serrated (size 6, For post space 3)	1.40 ± 0.05	1.50 ± 0.05	0.10 ± 0.05 (100)

**Table 4:** Descriptive statistics with mean, standard deviation, and ANOVA results for the tensile bond strength.

Groups	Material Groups	N	^a^ Mean	Std. Deviation	95% Confidence Interval for Mean		Minimum	Maximum	^b^ Anova *p*-Value
					Lower Bound	Upper Bound			
Group A	Space 1	10	170.65	7.93	164.97	176.33	154.34	180.57	0.000
	Space 2	10	194.35	11.60	186.04	202.65	179.45	213.52	
	Space 3	10	281.42	60.36	238.24	324.60	200.21	345.88	
Group B	Space 1	10	131.24	12.19	122.51	139.96	109.57	150.77	0.000
	Space 2	10	150.06	10.66	142.43	157.69	136.17	167.75	
	Space 3	10	179.26	7.13	174.16	184.36	167.30	190.47	
Group C	Space 1	10	89.75	8.83	83.43	96.06	78.92	100.28	0.000
	Space 2	10	117.27	14.19	107.12	127.42	97.01	142.18	
	Space 3	10	155.01	26.44	136.09	173.92	128.56	215.87	

^a^ Mean tensile strength was measured in Megapascals (MPa). ^b^ The *p* value was significant at *p* < 0.05. Groups A and C: Space 1—size 4, Space 2—size 5, Space 3—size 6. Group B: Space 1—size 0, Space 2—size 1, Space 3—size 2.

**Table 5:** Multiple Comparisons and mean differences of the test specimens with different post spaces by post-hoc Tukey HSD test.

Dependent Variable	Post Space	Comparison	Mean Difference	Sig.	95% Confidence Interval	
					Lower Bound	Upper Bound
Group A	Space 1	Space 2	−23.69	0.316	−63.37	15.98
		Space 3	−110.76 ^a^	0.000	−150.44	−71.08
	Space 2	Space 1	23.69	0.316	−15.98	63.37
		Space 3	−87.07 ^a^	0.000	−126.75	−47.39
	Space 3	Space 1	110.76 ^a^	0.000	71.08	150.44
		Space 2	87.07 ^a^	0.000	47.39	126.75
Group B	Space 1	Space 2	−18.82 ^a^	0.001	−30.15	−7.49
		Space 3	−48.02 ^a^	0.000	−59.35	−36.69
	Space 2	Space 1	18.82 ^a^	0.001	7.49	30.15
		Space 3	−29.20 ^a^	0.000	−40.53	−17.86
	Space 3	Space 1	48.02 ^a^	0.000	36.69	59.35
		Space 2	29.20 ^a^	0.000	17.86	40.53
Group C	Space 1	Space 2	−27.52 ^a^	0.006	−47.54	−7.49
		Space 3	−65.26 ^a^	0.000	−85.28	−45.23
	Space 2	Space 1	27.52 ^a^	0.006	7.49	47.54
		Space 3	−37.74 ^a^	0.000	−57.76	−17.71
	Space 3	Space 1	65.26 ^a^	0.000	45.23	85.28
		Space 2	37.74 ^a^	0.000	17.71	57.76

^a^ The mean difference was significant at *p* < 0.05. Groups A and C: Space 1—size 4, Space 2—size 5, Space 3—size 6. Group B: Space 1—size 0, Space 2—size 1, Space 3—size 2.

**Table 6:** Comparison of the tensile bond strength of the tested post materials with different post spaces (N = 90).

Post Space	Material Groups	N	^a^ Mean	Std. Deviation	95% Confidence Interval for Mean		Minimum	Maximum	^b^ Anova *p*-Value
					Lower Bound	Upper Bound			
Space 1	Group A	10	170.65	7.93	164.97	176.33	154.34	180.57	0.000
	Group B	10	131.24	12.19	122.51	139.96	109.57	150.77	
	Group C	10	89.75	8.83	83.43	96.06	78.92	100.28	
Space 2	Group A	10	130.55	34.90	117.51	143.58	78.92	180.57	0.000
	Group B	10	194.35	11.60	186.04	202.65	179.45	213.52	
	Group C	10	150.06	10.66	142.43	157.69	136.17	167.75	
Space 3	Group A	10	117.27	14.19	107.12	127.42	97.01	142.18	0.000
	Group B	10	153.89	34.22	141.11	166.67	97.01	213.52	
	Group C	10	281.42	60.36	238.24	324.60	200.21	345.88	

^a^ Mean tensile strength was measured in Megapascals (MPa). ^b^ The *p* value was significant at *p* < 0.05. Groups A and C: Space 1—size 4, Space 2—size 5, Space 3—size 6. Group B: Space 1—size 0, Space 2—size 1, Space 3—size 2.

## Data Availability

Data is available on request from the corresponding author.
